# Interaktives Lernen: Ethik Online im Medizinstudium

**DOI:** 10.1007/s00481-021-00647-w

**Published:** 2021-07-16

**Authors:** Dennis Krämer, Stefan Schulz, Joschka Haltaufderheide, Esther Braun, Matthé Scholten, Jochen Vollmann

**Affiliations:** Institut für Medizinische Ethik und Geschichte der Medizin, Ruhr-Universität Bochum, Markstraße 258a, 44799 Bochum, Deutschland

## Überblick

Das Gebot des „Social Distancing“ während der COVID-19-Pandemie hat eine Revision der bisherigen Lehr-Lern-Settings an Universitäten erforderlich gemacht. Als positiven Nebeneffekt hat die begegnungsarme Kommunikation einen Innovationsschub in puncto digitale Lehre ausgelöst und diente vielen Dozierenden als Anlass, neue Formate zu erproben. Vor diesem Hintergrund hat das Institut für Medizinische Ethik und Geschichte der Medizin der Medizinischen Fakultät der Ruhr-Universität Bochum ein neues Lehrkonzept für den Pflicht-Querschnittsbereich Geschichte, Theorie, Ethik der Medizin (GTE) im Studium der Humanmedizin entwickelt.^1^ Im Folgenden geben wir einen Einblick in die Erwartungen des Teams, die Herausforderungen, die sich bei der Umsetzung gestellt haben, und die Resonanz der Studierenden.

## Von der analogen zur digitalen Lehre

Der hier vorgestellte GTE-Kurs ist das Resultat eines dreisemestrigen Entwicklungsprozesses, in dem die sechsköpfige Autor*innengruppe in regelmäßigen Treffen die laufende Lehre evaluiert und Erfahrungen ausgetauscht hat, um auf dieser Grundlage eine konzeptionelle Umsetzung zu planen. Die Herausforderung bestand darin, ein Format zu entwickeln, um die Risiken eines monotonen, Bildschirm-fixierten und sich aus der räumlichen Distanz ereignenden Unterrichts zu minimieren. Drei Ziele standen dabei im Zentrum:

1. *Integration der Lehrinhalte*, wobei ethische, historische und rechtliche Aspekte möglichst plastisch an konkreten Fallbeispielen veranschaulicht werden sollten,
2. *Realisierung wechselseitiger Kommunikation*, das heißt digitale Lösungen zu erarbeiten, um kollaborativ zu arbeiten, Nachfragen zu stellen oder auch Feedbacks abzugeben,
3. *Arbeiten in vertrauter Atmosphäre* mit klar kommunizierten, verlässlichen Regeln, visueller Teilnahme aller Studierenden an den synchronen Veranstaltungen (keine „schwarzen Kacheln“) und dem Einsatz bekannter E‑Learning-Tools, damit die Studierenden angesichts der ohnehin bestehenden großen Umstellungen in der Lehre nicht durch Einsatz exotischer Tools oder Plattformen überfordert werden sollten.

## Vorstellung des Curriculums

**Figure 1:**
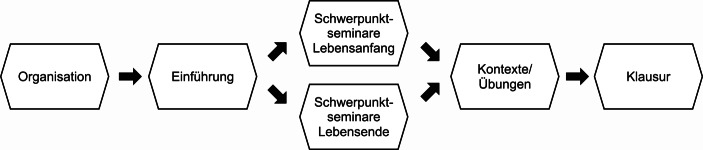

Zur Umsetzung entschieden wir uns dazu, bereits existierende Elemente aus der Präsenz- und Distanzlehre zu kombinieren und auf vertraute Plattformen sowie möglichst wenige Tools zurückzugreifen. Um Anteile einer synchronen und asynchronen Lehre zu verzahnen, griffen wir auf die fachübergreifend in der Bochumer Lehre verwendete Lernplattform „Moodle“ zurück: Diese ermöglicht den direkten Zugriff auf heterogene Quellen wie Folien, Dokumente, Feedbacks, Links oder Quizze und lässt sich zugleich zur Kommunikation von Sprechstunden, Klausurterminen und Veranstaltungsplänen nutzen. Das Videokonferenzsystem „Zoom“ mit seinen Funktionen des Video- und Textchats, Datenaustauschs sowie der Erstellung von Ad-Hoc-Gruppen wurde als Plattform für die Kommunikation in der synchronen Lehre eingesetzt. Strukturell haben wir den entwickelten GTE-Kurs in fünf aufeinander aufbauende Module unterteilt, die die Studierenden auf die Klausur vorbereiten und ihnen zugleich die Möglichkeit einräumen, eigene inhaltliche Akzente zu setzen. Innerhalb der jeweiligen Module wurden synchrone und asynchrone Elemente im Wechsel eingesetzt. (Abb. 1).

Der Ablauf gestaltet sich wie folgt: Vor der Einführungsveranstaltung werden die Studierenden in einen vorbereiteten Moodlekurs eingeladen, in dem alle relevanten Materialien und Informationen vorliegen. Auch besteht hier ein Angebot an weiteren Quellen (Links, Kommentare, weiterführende Literatur etc.), um Inhalte im Selbststudium zu vertiefen. Die einzelnen Module schließen mit einem freiwilligen und anonymen Online-Feedback ab, in dem die Studierenden die Gelegenheit erhalten, Verbesserungsvorschläge, Ideen und Wünsche für die Lehre mitzuteilen. Die studentischen Feedbacks hatten in den vergangenen Semestern wesentlichen Einfluss auf die Aktualisierung der Lehrveranstaltungen.

Die eigentliche Lehre beginnt mit einer sich über zwei Sitzungen erstreckenden Einführung in Medizingeschichte und Medizinethik, die in Moodle vorbereitet und via Zoom synchron übertragen wird. Anschließend gehen die Studierenden in eine selbstgewählte Vertiefungsphase: An den Themen „Lebensanfang“ bzw. „Lebensende“ erarbeiten sie zunächst im Selbststudium zentrale ethische Konzepte. In den anschließenden „Schwerpunktseminaren“ werden diese mit den Dozierenden synchron über Zoom diskutiert und gemeinsam vertieft. Zudem erfolgt eine Bearbeitung konkreter Fallbeispiele in Kleingruppen, die über Breakout-Räume realisiert wird. Die Fallbeispiele werden dabei in Form einer „ethischen Fallbesprechung“ aufgearbeitet, die es den Studierenden ermöglicht, sich normativen Fragen in praxisnaher Weise anzunähern. Im Zentrum der Schwerpunktseminare „Lebensanfang“ steht die Erarbeitung grundlegender Kenntnisse über ethische und rechtliche Aspekte von Schwangerschaftsabbruch, pränataler Gendiagnostik, IVF, PID und Embryonenschutzgesetz. In den Schwerpunktseminaren „Lebensende“ erarbeiten die Studierenden ein grundlegendes Wissen über ethische und rechtliche Aspekte von informierter Einwilligung, Einwilligungsfähigkeit, stellvertretende Entscheidungsfindung sowie Entscheidungen am Lebensende. Unter dem Schlagwort „Kontexte“ werden die jeweiligen Schwerpunktthemen im historischen Kontext synchron diskutiert und asynchron vertieft. Die anschließenden „Übungen“ dienen dazu, die Inhalte der jeweils nicht besuchten Schwerpunktseminare in komprimierter Form wiederzugeben und den Studierenden mit Blick auf die anstehende Klausur eine Möglichkeit zu geben, offene Punkte anzusprechen.

## Feedback und Erfolgskontrolle

Wie haben die Studierenden die Umstellung auf die Onlinelehre wahrgenommen?

Erste Einblicke in die Feedbacks des aktuellen Semesters zeigen, dass das neue Konzept sehr positiv beurteilt wird: Die Studierenden fühlen sich gut aufgehoben in einer digitalen Arbeitsatmosphäre und schätzen insbesondere die Diskussionen und Struktur der Veranstaltung. In den Feedbacks finden sich entsprechende Wertschätzungen der „guten Diskussionskultur“, „offenen Diskussionen und Umfragen“ sowie Einschätzungen, dass die „Seminare sehr lebendig“ seien. Kritisiert werden Punkte wie die Kürze der Diskussionen („Debatte hätte länger sein können“) sowie die über Breakout-Räume organisierte Kleingruppenarbeit, was u. a. mit ungleichen Redeanteilen begründet wird („die Gruppenarbeit gestaltet sich leider etwas schwierig wenn man mit nicht allzu redseligen Kollegen in einen Breakout-Room kommt“).

Als abschließende Erfolgskontrolle werden traditionell Multiple Choice Klausuren eingesetzt, woran wir auch in der Corona-Krise festhielten. Bereits im Wintersemester 20/21 wurden die Klausuren bei identischem Umfang von 24 Fragen erstmals als Onlineklausur von zu Hause geschrieben. Auf Kontrollmaßnahmen wie Videoüberwachung und Ausweiskontrolle verzichteten wir. Stattdessen wählten wir ein Format, das die zu bearbeitenden Klausuraufgaben in randomisierter Reihenfolge abbildet und für die Beantwortung einen vorab definierten Zeitraum vorsieht; bei unserer GTE-Klausur insgesamt 40 min. Trotz der Umstellung konnten wir keine wesentlichen Notenverschiebungen verzeichnen: Durchfallquote, Notenverteilung und Anzahl der Teilnehmenden sind nahezu identisch. Auf der Grundlage dieser ersten positiven Erfahrungen haben wir uns entschieden, an einer digitalen GTE-Lehre festzuhalten und diese in den nächsten Jahren weiterzuentwickeln.

## Footnotes

1 Unser Institut ist für die GTE-Lehre an den Medizinischen Fakultäten der Ruhr-Universität Bochum und der Universität Duisburg-Essen verantwortlich. Der neue GTE-Kurs wurde für die Lehre an der Medizinischen Fakultät der Universität Duisburg-Essen entwickelt und dort im Sommersemester 2021 erstmals eingesetzt.

